# Absence of early metabolic response assessed by 18F-FDG PET/CT after initiation of antifibrotic drugs in IPF patients

**DOI:** 10.1186/s12931-019-0974-5

**Published:** 2019-01-15

**Authors:** Benjamin Bondue, Amélie Castiaux, Gaetan Van Simaeys, Céline Mathey, Félicie Sherer, Dominique Egrise, Simon Lacroix, François Huaux, Gilles Doumont, Serge Goldman

**Affiliations:** 1 Department of Respiratory Medicine, Erasme University Hospital, Université libre de Bruxelles (ULB), route de Lennik 808, 1070 Brussels, Belgium; 2 Department of Nuclear Medicine, Erasme University Hospital, Université libre de Bruxelles (ULB), route de Lennik 808, 1070 Brussels, Belgium; 3 Center for Microscopy and Molecular Imaging, Université libre de Bruxelles (ULB), rue Adrienne Bolland 8, 6041 Charleroi, Belgium; 4 Louvain Centre for Toxicology and Applied Pharmacology, Institut de Recherche Expérimentale et Clinique, Université catholique de Louvain, Avenue Hippocrate, 57 bte B1.57.06, 1200 Woluwe-Saint-Lambert, Belgium

**Keywords:** Idiopathic pulmonary fibrosis, IPF, PET/CT, Biomarker, Interstitial lung disease, ILD, Nintedanib, Pirfenidone

## Abstract

**Background:**

Idiopathic pulmonary fibrosis (IPF) is characterized by a progressive and irreversible respiratory failure. Non-invasive markers of disease activity are essential for prognosis and evaluation of early response to anti-fibrotic treatments.

**Objectives:**

The aims of this study were to determine whether fluorodeoxyglucose ([18F]-FDG) lung uptake is reduced after initiation of pirfenidone or nintedanib and to assess its possible use as a prognostic factor.

**Methods:**

[18F]-FDG PET/CT was performed in IPF patients and in a murine model of pulmonary fibrosis. PET/CTs were performed at day 8 and day 15 post-instillation of bleomycin in pirfenidone- or vehicule-treated mice. In IPF patients, PET-CT was performed before and 3 months after the initiation of pirfenidone or nintedanib.

**Results:**

In bleomycin-treated mice, pirfenidone significantly reduced the [18F]-FDG uptake compared to vehicule-treated mice at day 15 (*p* < 0.001), whereas no difference was observed at day 8 after bleomycin administration. In IPF patients, [18F]-FDG lung uptake before and after 3 months of treatment by nintedanib (*n* = 11) or pirfenidone (*n* = 14) showed no significant difference regardless the antifibrotic treatment. Moreover, no difference was noticed between patients with progressive or non-progressive disease at one year of follow up.

**Conclusions:**

Pirfenidone significantly reduces the lung [18F]-FDG uptake during the fibrotic phase in a mouse model of IPF. However, these preclinical data were not confirmed in IPF patients 3 months after the initiation of antifibrotic therapy. [18F]-FDG seems therefore not useful in clinical practice to assess the early response of IPF patients to nintedanib or pirfenidone.

**Electronic supplementary material:**

The online version of this article (10.1186/s12931-019-0974-5) contains supplementary material, which is available to authorized users.

## Background

Idiopathic pulmonary fibrosis (IPF) is a chronic, irreversible, and progressive fibrosing interstitial lung disease (ILD), of unknown aetiology, leading to a terminal respiratory insufficiency [1]. The prognosis for patients with IPF is poor with a median survival rate between 2 and 5 years [1, 2]. The etiology of IPF is unknown but a role of environmental and genetic factors is suspected, resulting in repeated injuries of type II alveolar epithelial cells (AECII) associated with an excessive and inappropriate fibrotic response [3]. Its clinical course is unpredictable with the majority of patients experiencing a slow progression over many years, while others show accelerated decline with or without acute exacerbations [1]. Identification and validation of predictive markers of the clinical evolution are therefore crucial.

Recent studies have analyzed the value of the 2-deoxy-2-(18F)fluoro-D-glucose positron emission tomography with computed tomography ([18F]-FDG PET/CT) in IPF. An increase in lung uptake of [18F]-FDG was observed in areas of reticular/honeycombing and ground-glass opacity on high-resolution CT (HRCT) [4] as well as in areas of normal pulmonary parenchyma [5]. Recent studies have also evaluated the prognostic value of the standardized uptake value (SUV) and other derived parameters in IPF patients [6–9]. Moreover Justet and colleagues demonstrated that SUVmean and SUVmax were correlated with the severity of the lung involvement as measured by a decline in forced vital capacity (FVC) and lung diffusion capacity for carbon monoxide (DLCO) and increased GAP score [8].

The increased [18F]-FDG uptake observed in IPF is a marker of aerobic glycolysis that has been demonstrated to increase in fibrotic lungs [10, 11]. However, the precise cell type involved is not known. Some evidence suggests that it may be directly related to fibroblasts and epithelial cells [10, 12–14]. The role of the fibroblastic activity on [18F]-FDG uptake in IPF is also supported by a preclinical study in a mouse model of pulmonary fibrosis showing [18F]-FDG uptake not only during the initial inflammatory phase of the bleomycin-induced lung injury but also in the latter fibrotic phase [15].

Two antifibrotic drugs are approved for the treatment of IPF, nintedanib and pirfenidone [16, 17]. Since IPF is a highly variable disease with patients having slow or rapid progression or experiencing acute exacerbations, biomarkers predicting the clinical evolution and the response to antifibrotic drugs are needed. For these reasons, the focus of the present study was to conduct a monocentric study evaluating changes in the [18F]-FDG lung uptake after the initiation of an antifibrotic therapy in a preclinical mouse model and in newly diagnosed human IPF patients. We have analyzed changes in SUVmean and SUVmax, but also in previously published derived parameters with prognostic significance, such as the metabolic lung volume (MLV), the total lung glycolysis (TLG), which is the product of MLV and SUVmean [8], and the target-to-background (SUVmax/ SUVmin) ratio (TBR) [6]. We have also analyzed the SUV corrected for lung density since changes in lung density, which characterize IPF lung, can significantly influence per se the FDG uptake. Indeed, even if tissue uptake remains constant, a higher tissue/air ratio results in higher SUV values. Pulmonary function impairment evolves in parallel with lung density, so any relationship between pulmonary function and [18F]-FDG uptake in an undifferentiated lung volume may be driven by density changes, regardless of the metabolic demands by the pulmonary cells involved in inflammatory or fibrotic processes. So, if [18F]-FDG PET/CT is used to assess the intrinsic activity of the disease (and not only its severity), the influence of lung density should be taken into consideration in the [18F]-FDG uptake evaluation ([18, 19], and unpublished data).

## Material and methods

### Preclinical study

#### Mice

Eight to ten weeks old C57BL/6 female mice (Charles River) were used throughout these studies. They were hosted and bred in a specific pathogen free environment with access to food and water ad libitum. All procedures were reviewed and approved by the local committee for animal welfare.

#### Lung fibrosis model and pirfenidone treatment

Lung fibrosis was induced by trans-oral administration of bleomycin. Mice were anesthetized with a mix of 1 mg ketamine (Ketalar, Ceva) and 0.2 mg xylazine (Rompun, Bayer AG). After visualisation of the trachea’s lumen, *trans-*oral instillation of 0.02 U of bleomycin (Sanofi Aventis) in 60 μL NaCl 0.9% or NaCl 0.9% only (controls) was performed into the trachea. Mice orally received twice daily either 200 mg/kg/dose of pirfenidone resuspended in 40 mg/mL carboxymethylcellulose (CMC) or CMC alone starting at the day of bleomycin administration. At day 8 or day 15, mice were used for PET imaging or sacrificed for further investigations by exsanguination under deep isoflurane-induced anesthesia (4% isoflurane in 1 L/min O_2_).

#### Quantitative assessment of lung fibrosis

Lung total collagen was estimated by measuring hydroxyproline (HPO), a specific component of collagen. Lungs were homogenized on ice with an Ultra-Turrax T25 homogenizer (Janke & Kunkel) and stored at − 80 °C. Whole lung homogenate was hydrolyzed in HCl 6 N at 108 °C during 24 h and HPO was quantified by high-performance liquid chromatography. Cytokine (IL-6) and chemokine (CCL2) levels were also measured in the supernatant of lung homogenates using ELISA kits (R&D Systems).

#### Bronchoalveolar lavage (BAL)

Bronchoalveolar lavages (BAL) were obtained at day 15 post bleomycin treatment by flushing the lungs with 0.8 mL sterile 0.9% NaCl, and differential cell counts were performed on cytospin preparations after Diff-Quick staining (Dade Behring).

#### [^18^F]-FDG PET-CT imaging

The metabolic activity was assessed with [18F]-FDG PET/CT imaging at 8 and 15 days post-bleomycin or saline instillation. Mice were fasted overnight. The [18F]-FDG was synthesized at the PET/Biomedical Cyclotron Unit of the Nuclear Medicine Department at ULB-Hôpital Erasme (Brussels, Belgium). Mice were injected intravenously with about 3.9 MBq (SD = 0.39) of [18F]-FDG and kept under isoflurane anesthesia for 10 min post-injection in order to limit tracer uptake within skeletal muscle and brown adipose tissue [20]. PET/CT imaging was performed under isoflurane anesthesia 60 min after [18F]-FDG injection using a preclinical PET-CT tomograph (nanoPET-CT, Mediso). PET emission images were recorded for 15 min in 3-to-1 coincidence mode in normal count rate. CT acquisition parameters were 55 kV for a tube current of 145 μA, 1100 ms per projection, 180 projections per rotation, pitch of 1, a frame binning of 2 by 2, and a cubic reconstructed voxel size of 212 μm. Scaled CT images were used to obtain CT-attenuation-corrected and scatter-corrected PET images. All PET images were also corrected for random counts, dead time and decay. The PET acquisitions were reconstructed using a fully 3-dimensional iterative OSEM reconstruction algorithm (4 iterations, 6 subsets, intermediate regularization setting, median filtering period defined from iteration counts). Three-dimensional spherical ROIs (12-pixel diameter) were drawn on pulmonary parenchyma in left and right lung using InVivoScope2.0 (inviCRO). Care was taken to exclude voxels close to the heart. Mean and maximal activity within each ROI was subsequently divided by the ratio A_0_ (injected activity decay-corrected at the start of the PET acquisition [Bq] to animal weight [g]) in order to provide a mean and maximal SUV (SUVmean and SUVmax respectively). For each mouse, SUV for right and left lungs were averaged before performing statistical analysis.

### Human study

#### Patients

Between 2013 and 2016, 25 IPF patients were prospectively included in the study. These patients were followed at the Erasme hospital until April 2018. An [18F]-FDG PET/CT scan was performed before and 3 months after the initiation of either pirfenidone or nintedanib. The diagnosis of IPF was established in accordance with the ATS/ERS/JRS/ALAT recommendations [1] through a multidisciplinary discussion involving pulmonologists, radiologists, internal medicine specialists (or rheumatologists) and pathologists experienced in the diagnosis of ILDs. Patients under immunosuppressive therapy were excluded, with the exception of those receiving a low dose of corticosteroids (4 mg or less of methylprednisolone or equivalent). Any suspicion or proved acute exacerbation of IPF defined according to a recent International Working Group Report [21] within three months prior to the inclusion in the study excluded the patient. The protocol has been approved by the Erasme hospital Ethics Committee (ref. P2016/427). Written informed consent for participation in the study was obtained from all the patients.

#### Clinical investigations

Information obtained included age, sex, tobacco exposure and treatments. For each patient, we analyzed the pulmonary function tests (PFT) at the time of PET/CT and then every three months for at least one year of follow-up. The following parameters were recorded: diffusing capacity of the lung for carbon monoxide (DLCO), forced vital capacity (FVC), and total lung capacity (TLC). A 6-min-walk test (6MWT) was performed at the time of PET/CT and one year later. For each patient, the GAP index was calculated as described by Ley and al [22].

.To assess the clinical evolution at one year, the slope of the FVC, TLC, and DLCO evolution was determined by a linear regression using least-squares method. This method was preferred to cope with the variability of PFT measurements performed routinely in clinical practice. The disease activity for each patient was further characterized as “progressive” or “non-progressive”. A patient was classified as having a progressive disease if any of the following change occurred during the one-year follow-up period: ≥10% absolute decrease from baseline in FVC, ≥ 15% absolute decrease from baseline in DLCO, lung transplantation or death related to IPF.

#### PET-CT imaging

All imaging examinations were performed using a dedicated PET/CT scanner (Gemini GS16P, Philips Medical Systems, Cleveland, OH, USA). All patients were fasted for at least 6 h before the examination. Blood glucose level before [18F]-FDG injection should not exceed 150 mg/dL. PET/CT images were obtained 60 min after injection of 300-370 MBq of [18F]-FDG. Low-dose CT was used for attenuation correction of the PET emission data. Three-dimensional regions of interest (ROI) were drawn on pulmonary parenchyma in the left and right lung using VivoQuant 2.5 (inviCRO, Boston, MA, USA). The quantitative FDG uptake in these regions was evaluated by SUVmean and SUVmax. These parameters were corrected for the tissue fraction, according to an already validated method (18,19, and unpublished data). Briefly, CT images were obtained for each patient to determine the lung density in Hounsfield units. Then, a coefficient k corresponding to the tissue fraction was calculated as follows and as previously published [18]:

$$ \mathrm{k}=\frac{\mathrm{H}{\mathrm{U}}_{\mathrm{Lung}}-\mathrm{H}{\mathrm{U}}_{\mathrm{Air}}}{\mathrm{H}{\mathrm{U}}_{\mathrm{Tissue}}-\mathrm{H}{\mathrm{U}}_{\mathrm{Air}}}, $$

Where HU_Lung_, HU_Tissue_ and HU_Air_ are the densities (in Hounsfield units) of lung, soft tissue and air, respectively. HU_Lung_ is determined on the CT, HU_Tissue_ is estimated at 50, and HU_Air_ is equal to − 1000 HU. A matrix containing all these tissue fractions can be created, which allows PET images to be corrected by dividing the SUV values in each voxel by the corresponding tissue fraction:

$$ \mathrm{SU}{\mathrm{V}}_{\mathrm{corr}}=\frac{\mathrm{SU}{\mathrm{V}}_{\mathrm{uncorr}}}{\mathrm{k}} $$

Additional quantitative parameters derived from PET images were calculated (TBR, MLV and TLG). TBR is defined as the ratio SUVmax/SUVmin, with SUVmin being the minimum SUV value within the anatomical pulmonary volume. To assess the MLV, we need to define a threshold of FDG uptake for selecting the reportable metabolically active ROI within the anatomical pulmonary volume. This threshold was defined from a group of 15 normal patients who had an FDG-PET scan but had no lung pathology. The demographic profile of this group was identical to that of the patients in the study: 10 males, 5 females, with an average age of 68.33 years ±2.33 years. In this group, the average SUVmean was 0.51 in the whole lungs, with an average SD of 0.22. As a result, we have chosen to set the lower threshold at a value of SUVmean + 2 SD = 0.95, a value that was rounded to 1. This value is in agreement with the threshold used by Justet et al. [8]. Finally, we quantified the total lung glycolysis (TLG), which is the product of MLV and SUVmean, because it reflects the overall FDG uptake in the metabolically relevant lung volume.

### Statistical analysis

Correlations analyses were performed using Pearson’s correlation tests. Multiple comparisons were performed using ANOVA tests with Tukey’s multiple comparisons test for post hoc analysis. According to the results of the D’Agostino & Pearson test used to assess the normality of the sample values, simple comparisons between two groups were tested by unpaired Student t tests or Mann-Whitney tests. Proportions were compared using the Chi^2^ test. Statistical analyses were performed using GraphPad Prism 6 (GraphPad Software, La Jolla, California, USA). For all tests, a *P*-value of less than 0.05 was considered statistically significant.

## Results

### Pirfenidone significantly reduces [18F]-FDG uptake in the bleomycin model of pulmonary fibrosis

**Fig. 1:**
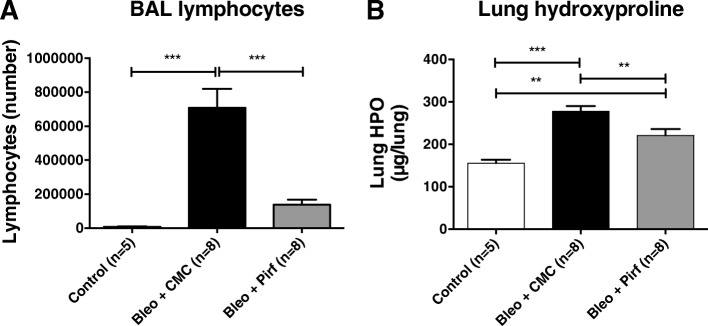
Pirfenidone reduces bleomycin-induced pulmonary fibrosis in mice. Groups of minimum five mice were used throughout these experiments. Mice were treated with either a saline sterile solution (control) or bleomycin (0.02 U) (Bleo). Bleomycin-treated mice orally received twice daily either 200 mg/kg/dose of pirfenidone resuspended in carboxymethylcellulose (Bleo + Pirf) or carboxymethylcellulose alone (Bleo + CMC). Saline-treated mice received carboxymethylcellulose (control). Mice were sacrificed at day 15 post-bleomycin administration for lymphocyte count in the BAL (**a**) and hydroxyproline measurement in the lungs (lung HPO) (**b**). Data are the mean ± SEM. Statistical analyses were performed by an ANOVA one-way test with Tukey’s test for comparisons between groups. **, *p* < 0.01; ***, *p* < 0.001

**Fig. 2:**
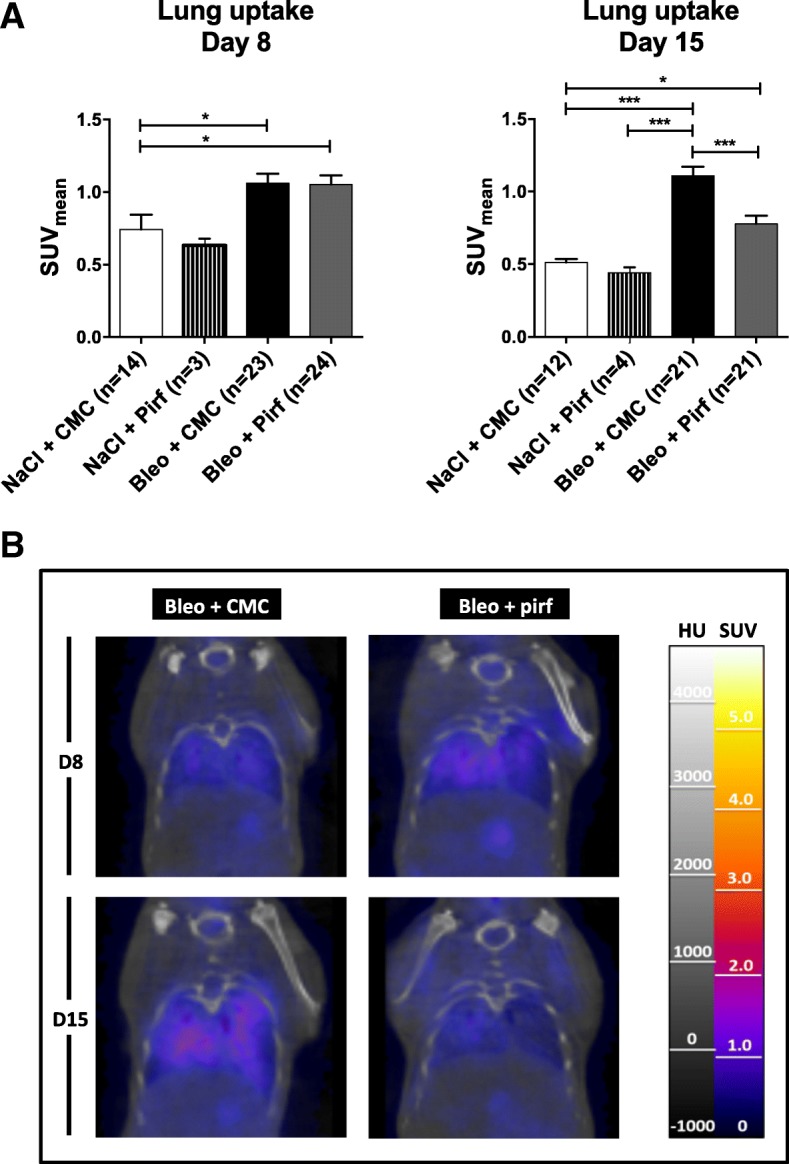
Pirfenidone reduces lung [18F]-FDG uptake during the fibrotic phase of the bleomycin-induced pulmonary fibrosis model. **a** Bleomycin (0.02 U)- and saline-treated mice orally treated with pirfenidone resuspended in carboxymethylcellulose (CMC) or CMC alone were used for in vivo imaging by [18F]-FDG PET/CT scan at day 8 and 15 after bleomycin instillation**.** Data shown corresponds to the lung SUVmean ± SEM and resulted from pooling of three Independent experiments. **b** Representative coronal sections obtained in bleomycin mice treated or not with pirfenidone at day 8 or 15 post instillation of bleomycin. Statistical analyses were performed by an ANOVA one-way test with Tukey’s test for comparisons between groups. *, *p* < 0.05; ***, *p* < 0.001

The bleomycin model of pulmonary fibrosis was used to assess any modification of the [18F]-FDG lung uptake in mice treated by pirfenidone. Pirfenidone diluted in CMC or CMC alone was administered twice daily. [18F]-FDG PET/CT scan was performed at day 8 and 15 days post- bleomycin-instillation and BAL and lungs were collected at day 15. Compared to control mice, bleomycin-treated mice showed a steep increase of lymphocyte counts in BAL (8 ± 4.10^3^ vs 708 ± 315.10^3^ respectively, mean ± SD, *p* < 0.001) and an increase of lung hydroxyproline (HPO) contents (156.0 ± 16.5 vs 278.0 ± 34.7 μg/lung respectively, mean ± SD, *p* < 0.001) (Fig. 1). Treatment of mice with pirfenidone induced a significant decrease (− 80%) of BAL lymphocyte numbers (137 ± 84.10^3^ vs 708 ± 315.10^3^, mean ± SD, p < 0.001), and lung hydroxyproline levels (221.1 ± 43.0 vs 278.0 ± 34.7 μg/lung, mean ± SD, *p* < 0.01). This corresponds to a − 20% absolute reduction or a − 47% relative reduction considering HPO level of NaCl-treated mice as reference. PET/CT data showed that administration of bleomycin significantly increased lung [18F]-FDG uptake at day 8 (0.74 ± 0.38 vs 1.06 ± 0.31, SUVmean ± SD for NaCl- vs bleomycin-treated mice; *p* < 0.05) and day 15 (0.51 ± 0.07 vs 1.11 ± 0.29, SUVmean ± SD for NaCl- vs bleomycin-treated mice; *p* < 0.001) (Fig. 2). Interestingly, in the bleomycin group, treatment with pirfenidone did not reduce the [18F]-FDG uptake in lung at day 8 (1.06 ± 0.31 vs 1.05 ± 0.32, SUVmean ± SD for bleomycin + CMC vs bleomycin + pirfenidone; *p* > 0.05), whereas a significant decrease was noted at day 15 (1.11 ± 0.29 vs 0.77 ± 0.25, SUVmean ± SD for bleomycin + CMC vs bleomycin + pirfenidone; *p* < 0.001).

### No significant [18F]-FDG uptake changes are observed in IPF patients following treatment with pirfenidone or nintedanib

A total of 25 patients were included in this study and their clinical characteristics are summarized in Table 1. The majority of the patients had a mild to moderate disease. 56% initiated a treatment by pirfenidone (*n* = 14) and 44% a treatment by nintedanib (*n* = 11). Patients were followed during one year and classified as having a progressive disease or not. The clinical characteristics of these two subgroups were compared and no significant differences were noticed (Table 1).

**Table 1:** Clinical characteristics of the IPF patients

Clinical characteristics	Patients (*n* = 25)	Progressive disease (*n* = 9)	Non-progressive disease (*n* = 15)	*p* values
Age (years, mean ± SD)	69 ± 8	67 ± 9	70 ± 8	NS
Gender				
Male % (n)	68 (17)	67 (6)	67 (10)	NS
Female % (n)	32 (8)	33 (3)	33 (5)	NS
Tabagism				
Non-smoker % (n)	12 (3)	11 (1)	13 (2)	NS
Active smokers % (n)	0 (0)	0 (0)	0	NS
past smokers % (n)	80 (20)	78 (7)	80 (12)	NS
Passive smoker % (n)	8 (2)	11 (1)	7 (1)	NS
Anti-fibrotic treatment				
Pirfénidone % (n)	56 (14)	67 (6)	46 (7)	NS
Nintedanib % (n)	44 (11)	33 (3)	54 (8)	NS
Pulmonary function tests				
FVC (l, mean +/− SD)	2,4 ± 0,8	2,2 ± 0,8	2,5 ± 0,7	NS
FVC (%, mean +/− SD	73,7 ± 20,2	63,6 ± 17,2	78,9 ± 20,6	NS
DLCO (ml/min/mmHg, mean +/− SD)	10,5 ± 3,5	9,3 ± 3,4	11,1 ± 3,4	NS
DLCO (%, mean +/− SD)	43,5 ± 12,0	38,3 ± 11,3	46,8 ± 11,5	NS
GAP score (n):				
Stage 1% (n)	28 (7)	11 (1)	40 (6)	NS
Stage 2% (n)	56 (14)	67 (6)	47 (7)	NS
Stage 3% (n)	16 (4)	22 (2)	13 (2)	NS

For 24 patients, a follow up was available to classify them as having a progressive or non-progressive disease (middle and right column respectively). NS = not significant (*p* < 0.05)

**Fig. 3:**
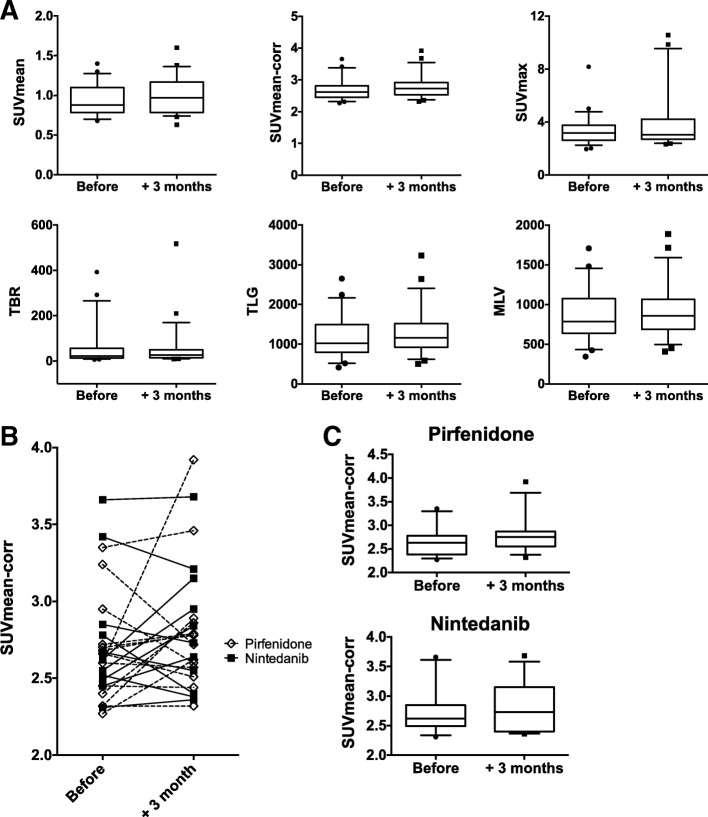
[18F]-FDG uptake changes before and 3 months after the initiation of antifibrotic drugs*.* **a** Different PET parameters were calculated (SUVmean, SUVmean corrected for the lung density - SUVmean-corr, SUVmax, metabolic lung volume -MLV, total lung glycolysis -TLG, target-to-background ratio -TBR) before and three months after the initiation of a treatment by pirfenidone or nintedanib. Data on graph correspond to the pooled results obtained with both antifibrotic drugs and consist of the median with whiskers corresponding to the percentile 10–90. **b** Individual changes in SUVmean-corr detailed for patients on pirfenidone (white boxes) and nintedanib (black squares). **c** The detailed changes in SUVmean-corr for patients on pirfenidone and nintedanib. Data on graph correspond to the median with whiskers corresponding to the percentile 10–90

**Fig. 4:**
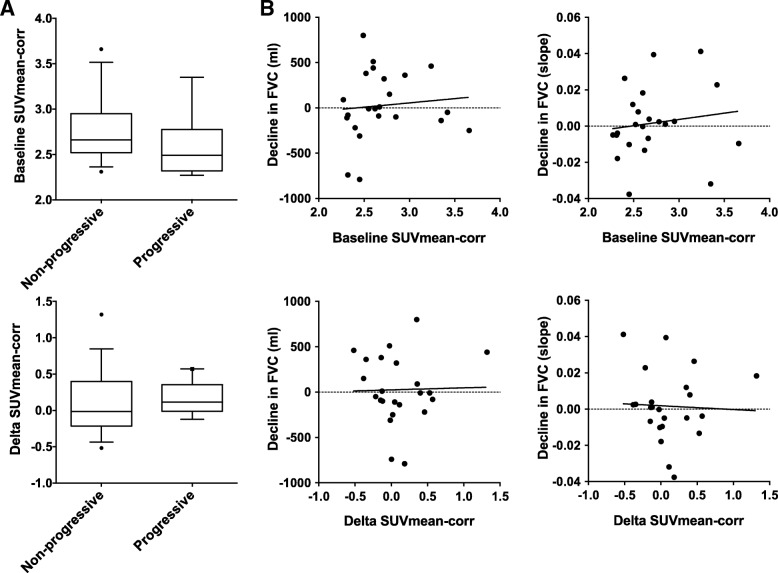
Prognostic value of baseline [18F]-FDG and Δ[18F]-FDG lung uptake. **a** Baseline SUVmean corrected for the lung density (SUVmean-corr) and change in SUVmean-corr three months after the initiation of the antifibrotic drug between patients having a progressive or a non-progressive disease after one year of follow-up. **b** Correlation between the decline in FVC (absolute value or using the slope of the overall evolution at one year obtained by linear regression using least-squares method) and baseline SUVmean-corr or change in SUVmean-corr three months after the initiation of the antifibrotic drug

[18F]-FDG lung uptake changes between baseline values and those obtained 3 months after the initiation of an antifibrotic therapy (Δ[18F]-FDG) were analyzed. The [18F]-FDG lung uptake was assessed using standard PET parameters such as SUVmean and SUVmax (both corrected or not for the lung density) but also using parameters known to have some prognostic value (MLV, TLG, and TBR). None of these PET parameters showed significant difference three months after the initiation of an antifibrotic treatment, regardless of the nature of the antifibrotic drug (pirfenidone or nintedanib) (*p* > 0.05) (Fig. 3 and Additional file 1 Figure S1). Next, we tested the prognostic value of the baseline [18F]-FDG lung uptake and Δ[18F]-FDG lung uptake. None of the analyzed PET parameters showed significant difference between progressive or non-progressive patients, considering either the baseline [18F]-FDG lung uptake or Δ[18F]-FDG lung uptake (Fig. 4a and Additional file 1 Figure S2; p > 0.05).

To assess the clinical evolution of the patients at one year, changes in FVC, TLC, and DLCO were analyzed. We used both absolute changes in FVC at one year and the overall evolution of these parameters by calculating, using least-squares method, their respective slope obtained by linear regression of all their values obtained during one year. No correlation between these functional parameters and the Δ[18F]-FDG lung uptake was found (*p* < 0.05) (Fig. 4b and Table 2). No correlation was also noticed between baseline SUVmean-corr and changes in FVC (Fig. 4b). Similarly, no correlation was found between 6MWT data (walk distance and oxygen saturation at the end of the test) and the Δ[18F]-FDG lung uptake at 3 months (Table 2).

**Table 2:** Correlations between changes in [18F]-FDG lung uptake after 3 months of antifibrotic treatment and the evolution of functional parameters

Correlation test (Pearson)				∆SUVmean	∆SUVmax	∆SUVmean-corr	∆TBR	∆TLG	∆MLV
Lung function tests	Evolution at one year (slope of the least squares)	FVC	r	−0.15	−0.21	−0.04	0.15	−0.20	−0.18
			p	0.46	0.31	0.87	0.47	0.34	0.38
		TLC	r	0.01	0.08	0.13	0.09	0.094	0.12
			p	0.97	0.69	0.54	0.68	0.65	0.57
		DLCO	r	−0.14	0.10	−0.11	0.18	−0.09	− 0.06
			p	0.49	0.61	0.59	0.39	0.63	0.78
6-MWT	Δ SaO_2_ post-exercise between baseline and after one year		r	−0.15	−0.31	−0.02	0.13	− 0.20	−0.20
			p	0.56	0.24	0.95	0.64	0.47	0.45
	Δ traveled distance between baseline and after one year		r	−0.25	−0.05	−0.08	0.22	− 0.10	−0.08
			p	0.37	0.86	0.77	0.43	0.74	0.78

Correlation tests were performed between the Δ[18F]-FDG uptake after three months of treatment and the evolution of lung function tests (FVC, TLC, and DLCO) and the evolution of the six-minutes walking test (6-MWT). r: correlation coefficient, p: p value

## Discussion

Idiopathic pulmonary fibrosis (IPF) is a debilitating, irreversible, fatal, fibrosing lung disease [1]. Value of metabolic evaluation by [18F]-FDG PET/CT has been studied in IPF patients [4–9]. An increased in lung uptake of [18F]-FDG is present in IPF lungs [4, 5] and SUV and other derived parameters have some prognostic value in these patients [6–9]. Two antifibrotics are currently available for the treatment of IPF patients, pirfenidone and nintedanib, which were approved in Europe in 2011 and 2015, respectively [16, 17]. However, no study has ever evaluated whether the administration of these drugs modifies the metabolic activity assessed by [18F]-FDG PET/CT. Therefore, the aim of our study was to evaluate if [18F]-FDG lung uptake, measured by PET/CT, was modified after the initiation of this therapy. Before conducting the clinical study on IPF patients, we used the bleomycin model of pulmonary fibrosis to test the hypothesis that the [18F]-FDG lung uptake was reduced when mice were treated by pirfenidone. Of note, no experiment was performed with nintedanib because this drug was not yet available in Belgium when this preclinical animal study was performed (early 2014). Pirfenidone was started immediately after administration of bleomycin to maximize the expected antififbrotic effect. This regimen was supported by previous studies evaluating the antifibrotic effect of pirfenidone in bleomycin-treated mice [23–25]. We observed that pirfenidone significantly reduces [18F]-FDG uptake in the lungs during the fibrotic phase of a bleomycin model (day 15) but not during the initial and more inflammatory phase (day 8). The lack of activity of pirfenidone on [18F]-FDG uptake at day 8 is important to consider since pirfenidone was expected to also present anti-inflammatory properties [23, 26]. Starting pirfenidone at the time of bleomycin administration, lung FDG uptake at day 8 could have been reduced if anti-inflammatory activity of the drug was substantial. But the lack of changes in FDG uptake at day 8 indicated that the anti-inflammatory properties of pirfenidone were too weak to significantly modify the severe inflammatory reaction observed in this early phase. Another possible explanation is that healing processes are already stimulated in mice treated with pirfenidone earlier than in control mice. The cells involved in these processes could participate to the global [18F]-FDG lung uptake. As a result, the total [18F]-FDG uptake by the lungs could therefore stay unchanged under therapy at the early stage of the model. Nevertheless, to better discriminate anti-inflammatory and antifibrotic activities of pirfenidone, preclinical experiments could be performed starting the pirfenidone after a latency period of 7 to 10 days. However, as such experimental design would not indisputably reproduce the clinical IPF situation in which nature and timing of the initial and repeated injuries remain undetermined, these complementary experiments were not performed and the human study initiated.

The human study was conducted in 25 IPF patients, all of whom had a [18F]-FDG PET/CT at baseline and three months after the initiation of pirfenidone or nintedanib treatment. Different PET parameters were analyzed, all described as having prognostic values in IPF [6, 8]. These include SUVmean, SUVmax, TBR, and TLG. SUVmean and SUVmax corrected for the lung density were also calculated and used throughout this study. Indeed, correction for lung density is important since lung density increases with the development of fibrotic tissue. This change needs to be taken into account when the [18F]-FDG uptake is used to evaluate pharmacological effect on the pulmonary tissue ([18, 19], and unpublished data). However, none of these parameters revealed significant differences in [18F]-FDG lung uptake between the baseline values and those obtained three months after the initiation of an antifibrotic treatment (either nintedanib or pirfenidone). We also analyzed whether the Δ[18F]-FDG uptake between the baseline values and those obtained three months after the initiation of the antifibrotic therapy could provide early prognostic information on the clinical evolution at one year. However, no significant difference was observed in the Δ[18F]-FDG uptake at three months between patients with progressive or non-progressive disease. Moreover, no correlation was found between changes in FVC at one year and both the baseline [18F]-FDG uptake or Δ[18F]-FDG uptake at three months.

This lack of prognostic role of the PET/CT could be explained by different factors. First of all, sensitivity of [18F]-FDG lung uptake is probably low to detect pharmacological effects since the signal remains relatively low even in severe IPF, as compared to the uptake detected in cancer. Second, the various contributions of immune cells, fibroblasts and myofibroblasts to the [18F]-FDG lung uptake limit the evaluation of drug effects on the sole fibrotic processes. Also, the lack of metabolic effects of the therapy found in our study may relate to the relatively weak antifibrotic effect of the drugs currently available for IPF. This last point is supported by a recent work made on transbronchial lung cryobiopsy in IPF patients under pirfenidone therapy showed no significant change in the number of fibroblastic foci or in tissue proliferation markers in biopsies obtained in patients under pirfenidone from their baseline value without treatment [27].

In our study, the PET/CT evaluation was performed before and 3 months after the initiation of the antifibrotic treatment. This methodological choice was supported by the early effect of pirfenidone on [18F]-FDG lung uptake in our mouse model. Indeed, we hypothesized that changes in the metabolic activity assessed by [18F]-FDG PET/CT in human could also be observed early after the initiation of the antifibrotic treatment as observed in mice. Moreover, the objective of the work was also to develop a metabolic assessment of drug efficacy at a time when switching to another therapy might increase chances of success. As a matter of fact, PET/CT evaluation performed at a later time point — at one year or later —, might provide other results. At one year, the predictive value of the PET/CT on the further response to the treatment would be reduced, and changes in FDG lung uptake from baseline would probably not only reflect the initiation of the antifibrotic treatment but also the fluctuant behaviour of IPF over time, with periods of rapid progression (related or not to acute exacerbations). Such changes in IPF behaviour could be associated with higher [18F]-FDG lung uptake. This has not been studied so far and could be another interesting research area not covered by the present work. By performing the PET/CT at 3 months, we hope to minimize the influence of these phenomena.

Our study presents several limitations. It consists of a monocentric study without any comparison with a placebo group. The natural variability as well as the evolution of [18F]-FDG lung uptake in untreated patients were therefore not taken into account in our analysis. In addition, the number of patients was relatively low and the follow-up period was limited to one year. The discrepancy between the human and animal studies is not fully understood. Firstly, the bleomycin model is an imperfect model of IPF. Fibrotic changes are reversible and followed a strong inflammatory reaction. Some pathological features of IPF — such as fibroblastic foci — also are lacking in the animal model. Secondly, pirfenidone has some anti-inflammatory properties and even if no difference in FDG uptake were seen at day 8, this could partly explain the later difference observed at day 14. Finally, inter- and intra-individual variability is much more important in human patients than in the murine model in which all animals are imaged at the precise same time point after the initiation of the pathologic process and after the start of the treatment; all mice also received the same dose of pirfenidone related to their weight, and all mice were genetically very close. The impact of inter-and intra-individual variability in human is particularly relevant in light of the relatively low values of SUV observed in IPF patients.

Despite our negative results, we are still convinced that specific markers of fibrogenesis able to predict the response to antifibrotic therapies are required in the future, specially if antifibrotic treatments may prove their efficacy in patients with progressive fibrosing interstitial lung disease other than IPF (PF-ILD). Indeed, this group of patients is heterogenous with often mixed inflammation and fibrosis. In these patients, it will be very important to better evaluate and discriminate inflammatory and fibrogenetic processes and to follow their respective changes over time. This would help the clinician to dynamically balance the choice between anti-inflammatory or antifibrotic drugs, opening an era of a more personalized medicine in PF-ILD. Candidate biomarkers predicting the response to nintedanib are presently investigated through the INMARK trial in IPF patients [28]. This trial evaluates the effect of nintedanib on the rate of changes in free-circulating biomarkers of ECM turnover as well as the correlation between these changes and disease progression. Alternatively, more specific PET/CT biomarkers of fibrogenesis could be developed. These comprise labelled peptides or antibodies targeting fibrotic pathways. Many candidates exist such as antibodies against CTGF, integrins, or lysyl oxydase. The PET/CT approach has the advantage not to be influenced by extra-pulmonary changes of the biomarker. Another advantage is to help the clinician to target a lung area to obtain a representative biopsy, if a pathological analysis is required for diagnostic purpose.

## Conclusions

Pirfenidone significantly reduces the [18F]-FDG uptake in the lungs during the fibrotic phase of the bleomycin mice model of IPF. However, these preclinical data were not confirmed in IPF patients three months after the initiation of an antifibrotic therapy. Moreover, changes in the [18F]-FDG lung uptake after the initiation of antifibrotic treatment have not clear prognostic value. Altogether, our data suggest that [18F]-FDG PET/CT has a limited role in detecting the early response to antifibrotic drugs. Therefore, this method does not currently appear to be suitable for the identification of patients who will benefit most from the therapy.

## Additional file

Additional file 1: **Figure S1.** PET parameters were calculated (SUVmean, SUVmax, metabolic lung volume -MLV, total lung glycolysis -TLG, target-to-background ratio -TBR) before and three months after the initiation of a treatment by pirfenidone (left column) or nintedanib (right column). Data on graph are the median with whiskers corresponding to the percentile 10–90. **Figure S2.** The different PET parameters related to the [18F]-FDG lung uptake (SUVmean, SUVmax, TBR, MLV, TLG) were measured at baseline and compared between progressive or non-progressive patients (left column). Their changes at three months following the initiation of the antifibrotic treatment was also calculated and also compared between progressive or non-progressive patients (right column). Data on graph are the median with whiskers corresponding to the percentile 10–90. (DOCX 59 kb)

## Abbreviations

6MWT: 6-minute-walk test
AECII: Type II alveolar epithelial cells
BAL: Bronchoalveolar lavage
CMC: Carboxymethylcellulose
CT: Computed tomography
DLCO: Diffusing capacity of the lung for carbon monoxide
FDG: 2-deoxy-2-fluoro-D-glucose
FVC: Forced vital capacity
HPO: Hydroxyproline
HU: Hounsfield units
ILD: Interstitial lung diseases
IPF: Idiopathic pulmonary fibrosis
MLV: Metabolic lung volume
PET: Positron emission tomography
PF-ILD: Progressive fibrosing interstitial lung disease
PFT: Pulmonary function test
ROI: Regions of interest
SD: Standard deviation
SEM: Standard error of the mean
SUV: Standardized uptake value
TBR: Target-to-background
TLC: Total lung capacity
TLG: Total lung glycolysis

## References

[1] Raghu G, Collard HR, Egan JJ, Martinez FJ, Behr J, Brown KK (2011). An official ATS/ERS/JRS/ALAT statement: idiopathic pulmonary fibrosis: evidence-based guidelines for diagnosis and management. Am J Respir Crit Care Med.

[2] Bjoraker JA, Ryu JH, Edwin MK, Myers JL, Tazelaar HD, Schroeder DR, Offord KP (1998). Prognostic significance of histopathologic subsets in idiopathic pulmonary fibrosis. Am J Respir Crit Care Med.

[3] Spagnolo P, Rossi G, Cavazza A (2014). Pathogenesis of idiopathic pulmonary fibrosis and its clinical implications. Expert Rev Clin Immunol.

[4] Groves AM, Win T, Screaton NJ, Berovic M, Endozo R, Booth H (2009). Idiopathic pulmonary fibrosis and diffuse parenchymal lung disease: implications from initial experience with 18F-FDG PET/CT. J Nucl Med Off Publ Soc Nucl Med.

[5] Win T, Thomas BA, Lambrou T, Hutton BF, Screaton NJ, Porter JC (2014). Areas of normal pulmonary parenchyma on HRCT exhibit increased FDG PET signal in IPF patients. Eur J Nucl Med Mol Imaging.

[6] Win T, Screaton NJ, Porter JC, Ganeshan B, Maher TM, Fraioli F (2018). Pulmonary 18F-FDG uptake helps refine current risk stratification in idiopathic pulmonary fibrosis (IPF). Eur J Nucl Med Mol Imaging.

[7] Lee EYP, Wong CS, Fung SL, Yan PK, Ho JCM (2014). SUV as an adjunct in evaluating disease activity in idiopathic pulmonary fibrosis - a pilot study. Nucl Med Commun.

[8] Justet A, Laurent-Bellue A, Thabut G, Dieudonné A, Debray M-P, Borie R (2017). [(18)F]FDG PET/CT predicts progression-free survival in patients with idiopathic pulmonary fibrosis. Respir Res.

[9] Nobashi T, Kubo T, Nakamoto Y, Handa T, Koyasu S, Ishimori T (2016). 18F-FDG uptake in less affected lung field provides prognostic stratification in patients with interstitial lung disease. J Nucl Med Off Publ Soc Nucl Med.

[10] Xie N, Tan Z, Banerjee S, Cui H, Ge J, Liu R-M (2015). Glycolytic reprogramming in myofibroblast differentiation and lung fibrosis. Am J Respir Crit Care Med.

[11] Maher TM (2015). Aerobic glycolysis and the Warburg effect. An unexplored realm in the search for fibrosis therapies?. Am J Respir Crit Care Med.

[12] Cho SJ, Moon J-S, Lee C-M, Choi AM, Stout-Delgado HW (2017). Glucose transporter 1-dependent glycolysis is increased during aging-related lung fibrosis, and Phloretin inhibits lung fibrosis. Am J Respir Cell Mol Biol.

[13] Mamchaoui K, Makhloufi Y, Saumon G (2002). Glucose transporter gene expression in freshly isolated and cultured rat pneumocytes. Acta Physiol Scand.

[14] Isobe K, Hata Y, Sugino K, Takai Y, Shibuya K, Homma S (2009). Usefulness of FDG-PET for diagnosis of lung cancer associated with interstitial pneumonia. Nihon Kokyūki Gakkai Zasshi J Jpn Respir Soc.

[15] Bondue B, Sherer F, Van Simaeys G, Doumont G, Egrise D, Yakoub Y (2015). PET/CT with 18F-FDG- and 18F-FBEM-labeled leukocytes for metabolic activity and leukocyte recruitment monitoring in a mouse model of pulmonary fibrosis. J Nucl Med Off Publ Soc Nucl Med.

[16] European Medicines Agency. Summary of Product Characteristics - Esbriet (pirfenidone). 2018. http://www.ema.europa.eu/ema/index.jsp?curl=pages/medicines/human/medicines/002154/human_med_001417.jsp&mid=WC0b01ac058001d124 (Accessed April 24 2018).

[17] European Medicines Agency. Summary of Product Characteristics - Ofev (nintedanib). 2017.http://www.ema.europa.eu/docs/en_GB/document_library/EPAR_Product_Information/human/003821/WC500182474.pdf (Accessed March 27 2018).

[18] Lambrou T, Groves AM, Erlandsson K, Screaton N, Endozo R, Win T (2011). The importance of correction for tissue fraction effects in lung PET: preliminary findings. Eur J Nucl Med Mol Imaging.

[19] Holman BF, Cuplov V, Millner L, Hutton BF, Maher TM, Groves AM (2015). Improved correction for the tissue fraction effect in lung PET/CT imaging. Phys Med Biol.

[20] Fueger BJ, Czernin J, Hildebrandt I, Tran C, Halpern BS, Stout D (2006). Impact of animal handling on the results of 18F-FDG PET studies in mice. J Nucl Med.

[21] Collard HR, Ryerson CJ, Corte TJ, Jenkins G, Kondoh Y, Lederer DJ (2016). Acute exacerbation of idiopathic pulmonary fibrosis. An international working group report. Am J Respir Crit Care Med.

[22] Ley B, Ryerson CJ, Vittinghoff E, Ryu JH, Tomassetti S, Lee JS (2012). A multidimensional index and staging system for idiopathic pulmonary fibrosis. Ann Intern Med.

[23] Iyer SN, Hyde DM, Giri SN (2000). Anti-inflammatory effect of pirfenidone in the bleomycin-hamster model of lung inflammation. Inflammation.

[24] Inomata M, Kamio K, Azuma A, Matsuda K, Kokuho N, Miura Y (2014). Pirfenidone inhibits fibrocyte accumulation in the lungs in bleomycin-induced murine pulmonary fibrosis. Respir Res.

[25] Song X, Yu W, Guo F (2018). Pirfenidone suppresses bleomycin-induced pulmonary fibrosis and periostin expression in rats. Exp Ther Med.

[26] Oku H, Shimizu T, Kawabata T, Nagira M, Hikita I, Ueyama A, Matsushima S, Torii M, Arimura A (2008). Antifibrotic action of pirfenidone and prednisolone: different effects on pulmonary cytokines and growth factors in bleomycin-induced murine pulmonary fibrosis. Eur J Pharmacol.

[27] Ronan N, Bennett DM, Khan KA, McCarthy Y, Dahly D, Bourke L, Chelliah A, Cavazza A, O'Regan K, Moloney F, Plant BJ, Henry MT. Tissue and Bronchoalveolar lavage biomarkers in idiopathic pulmonary fibrosis patients on Pirfenidone. Lung. 2018. 10.1007/s00408-018-0140-8. 10.1007/s00408-018-0140-8 30066212

[28] Maher TM, Stowasser S, Nishioka Y, White ES, Cottin V, Noth I (2018). Investigating the effects of nintedanib on biomarkers of extracellular matrix turnover in patients with IPF: design of the randomised placebo-controlled INMARK®trial. BMJ Open Respir Res.

