# Supplementary material for: Absence of early metabolic response assessed by 18F-FDG PET/CT after initiation of antifibrotic drugs in IPF patients
Source: Respir Res. 2019 Jan 15;20:10. doi: 10.1186/s12931-019-0974-5 (PMC6334423; doi:10.1186/s12931-019-0974-5)
Supplement: Supplementary file 1 — Figure S1. PET parameters were calculated (SUVmean, SUVmax, metabolic lung volume -MLV, total lung glycolysis -TLG, target-to-background ratio -TBR) before and three months after the initiation of a treatment by pirfenidone (left column) or nintedanib (right column). Data on graph are the median with whiskers corresponding to the percentile 10–90. Figure S2. The different PET parameters related to the [18F]-FDG lung uptake (SUVmean, SUVmax, TBR, MLV, TLG) were measured at baseline and compared between progressive or non-progressive patients (left column). Their changes at three months following the initiation of the antifibrotic treatment was also calculated and also compared between progressive or non-progressive patients (right column). Data on graph are the median with whiskers corresponding to the percentile 10–90. (DOCX 59 kb) [file 12931_2019_974_MOESM1_ESM.docx]

**Supplemental data**

**Figure S1**

**Figure S1.** PET parameters were calculated (SUVmean, SUVmax, metabolic lung volume -MLV, total lung glycolysis -TLG, target-to-background ratio -TBR) before and three months after the initiation of a treatment by pirfenidone (left column) or nintedanib (right column). Data on graph are the median with whiskers corresponding to the percentile 10-90.

**Figure S2.** The different PET parameters related to the [18F]-FDG lung uptake (SUVmean, SUVmax, TBR, MLV, TLG) were measured at baseline and compared between progressive or non-progressive patients (left column). Their changes at three months following the initiation of the antifibrotic treatment was also calculated and also compared between progressive or non-progressive patients (right column). Data on graph are the median with whiskers corresponding to the percentile 10-90.
